# Protective Effects of Ginkgolide on a Cellular Model of Alzheimer’s Disease via Suppression of the NF-*κ*B Signaling Pathway

**DOI:** 10.1007/s12010-022-03828-5

**Published:** 2022-02-07

**Authors:** Tian-Tong Niu, He Yin, Bao-Lei Xu, Ting-Ting Yang, Hui-Qin Li, Yi Sun, Guang-Zhi Liu

**Affiliations:** 1 Department of Neurology, Beijing Anzhen Hospital, Capital Medical University, Beijing, 100029 China; 2 Research & Development Centre of Chengdu Baiyu Pharmaceutical Co., Ltd, Chengdu, 610041 China

**Keywords:** Alzheimer’s disease, Bilobalide, Ginkgolide, Ginkgolide B, Neuroinflammation, NF-κB signaling pathway

## Abstract

NF-κB signaling has been reported to play a key regulatory role in the pathogenesis of Alzheimer’s disease (AD). The purpose of this study is to investigate the effects of ginkgolide on cell viability in an AD cellular model involving an APP/PS1 double gene-transfected HEK293 cell line (APP/PS1-HEK293) and further explore the mechanisms of action related to NF-*κ*B signaling. The optimal time point and concentration of ginkgolide for cell proliferation were screened using a cell counting kit-8 assay. Based on the results, an *in vitro* study was performed by co-culture of APP/PS1-HEK293 with different dosages of ginkgolide, followed by an enzyme-linked immunosorbent assay to measure the levels of supernatant tumor necrosis factor (TNF)-α, interleukin (IL)-1β and IL-6, as well as western blotting and real-time polymerase chain reaction to detect intracellular protein and mRNA expression of NF-*κ*B p65, I*κ*Ba, Bcl-2, and Bax. APP/PS1-HEK293 cells exhibited the highest cell viability at a concentration of 100 µg/ml after 48 h of treatment with ginkgolide. The supernatant levels of TNF-α, IL-1β, and IL-6 in the high-dosage ginkgolide-treated groups were lower than those in the control group. Compared with the control group, there were decreased intracellular protein and mRNA expression of NF-*κ*B p65 and Bax, but increased protein and mRNA expression of I*κ*Ba in both high-dosage and low-dosage groups. Ginkgolide may enhance cell viability, indicative of its neuroprotective effects on AD, at least partially via suppression of the NF-*κ*B signaling pathway involving anti-apoptosis and anti-inflammation mechanisms. Therefore, ginkgolide might be a promising therapeutic agent against AD.

**Graphical abstract:**

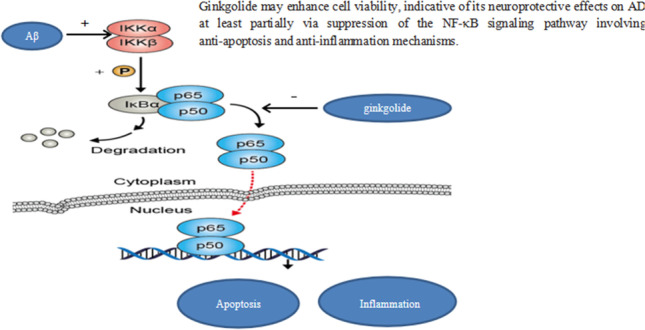

**Supplementary Information:**

The online version contains supplementary material available at 10.1007/s12010-022-03828-5.

## Introduction

As one of the most prevalent neurodegenerative disorders, Alzheimer’s disease (AD) is characterized by irreversible cognitive impairment and memory loss, with the accumulation of amyloid-beta (Aβ) and neurofibrillary tangles, which represent typical pathological features [1]. Increasing evidence has substantiated that the Aβ-induced inflammatory response plays a crucial role in the neurodegenerative process of AD, and Aβ-mediated neuroinflammation is predominantly regulated via the nuclear factor kappa B (NF-*κ*B) signaling pathway [2–4]. As a ubiquitously expressed transcription factor in eukaryotic cells of the nervous system, NF-*κ*B family transcription factors function as master regulators of immune development, immune response, inflammation, cancer, and apoptosis [5, 6]. Such activities are mediated through homo- or heterodimerization of NF-*κ*B subunits RelA/p65, RelB, c-Rel, p50, and p52, of which RelA/p65 is the most abundant subunit [7]. Proteins of the inhibitory *κ*B (I*κ*B) family function as inhibitors and regulators of NF-*κ*B activity. Members of the I*κ*B family include the classical I*κ*B proteins (I*κ*Bα, I*κ*Bβ, and I*κ*Bε), NF-*κ*B precursor proteins (p100 and p105), and nuclear IκBs (I*κ*Bζ, Bcl-3, and I*κ*BNS). Upon stimulation of innate immunity receptors, such as toll-like receptors and the cytokine receptor tumor necrosis factor receptor superfamily, a series of membrane-proximal events cause the activation of I*κ*B kinase (IKK). Phosphorylation of I*κ*Bs contributes to their proteasomal degradation, the release of NF-*κ*B for nuclear translocation, and activation of gene transcription, consequently leading to inflammation and immune response [8, 9]. Recent studies have confirmed that NF-*κ*B signaling has a key regulatory role in the pathogenesis of AD, and hence, it has been considered a compelling target for therapeutic intervention [10, 11].

Currently, both cholinesterase inhibitors (donepezil, carbazatin, and galanthamine) and N-methyl-D-aspartate receptor antagonists (memantine) are recommended to treat AD. However, these medications are not ideal because they temporarily ameliorate the symptoms of dementia without halting the progression of the disorder and produce remarkable adverse effects after long-term use. Although numerous new agents for the treatment of AD have been developed, such as aducanumab and crenezumab [12], they have not been fully implemented in clinical practice because of a lack of definitive therapeutic effects. GV-971, a marine algae-derived oral oligosaccharide, has been approved for clinical use, but its efficacy remains disputable [13]. In the past decade, botanical preparations with multi-target treatment and high-level safety have become a new trend in the research and development of therapeutic drugs for AD. Various *in vivo* and *in vitro* studies have reported the interventional effects of botanicals, such as resveratrol, *Rhodiola sachalinensis*, curcumin, and natural polyphenols on AD, suggesting their therapeutic potential for the prevention and treatment of AD [14–17].

As a botanical agent, Ginkgo biloba (GB) extract has been widely used to treat cerebrovascular diseases because of its multiple biological and pharmacological activities, such as antioxidative, anti-inflammatory, and anti-allergic effects as well as free radical scavenging and platelet aggregation suppression [18, 19]. Interestingly, several recent studies have revealed that GB extracts also exhibited certain therapeutic effects on dementia. Given these findings, GB extract has been recommended for the treatment of AD patients, especially for those who have failed to benefit from other treatments [20]. Currently, the international standard extract of GB is EGb 761, produced according to the German Schwabe patent process. EGb 761 is a well-defined plant extract product of Ginkgo biloba leaves. The extract contains two main active substances: flavonoid glycosides (24-26%) and terpene lactones (6–8%) consisting of ginkgolides A, B, C, and bilobalide [21]. Furthermore, based on technological advances, a new product of ginkgolide (Baiyu®), which is composed of ginkgolide ABCJ and bilobalide, has been recently developed and approved for the treatment of ischemic cerebrovascular disease. However, its therapeutic efficacy in the context of AD remains unclear. Hence, we performed a preliminary study on the effects of post-treatment withginkgolide (Baiyu®) and its components (ginkgolide B and bilobalide) on cell viability in an AD cellular model involving an APP/PS1 double gene-transfected HEK293 cell line (APP/PS1-HEK293), in order to initially assess the efficacy of this agent in the cell model, and further explored the related mechanisms of action.

## Materials and Methods

### Reagents

Ginkgolide (Baiyu®) (Catalog Number: Z20110035), bilobalide (Catalog Number: 20190730), and ginkgolide B (Catalog Number: 20170324) were kindly provided by Baiyu Pharmaceutical Co. Ltd (Chengdu, China). Dulbecco’s modified Eagle’s medium (DMEM) and fetal bovine serum were purchased from Gibco (Grand Island, NY, USA). The cell counting kit-8 (CCK-8) was purchased from Tongren Company (Japan). Human tumor necrosis factor (TNF)-α enzyme-linked immunosorbent assay (ELISA) kit, human interleukin (IL)-6 ELISA kit, and human IL-1β ELISA kit were purchased from eBioscience (San Diego, CA, USA). RIPA total protein extraction kit, phenylmethylsulfonyl fluoride, and bicinchoninic acid (BCA) protein assay kits were purchased from Sigma (St. Louis, MO, USA). Goat anti-rabbit IgG (H+L) HRP and goat anti-rabbit IgG (H+L) HRP were purchased from Jackson ImmunoResearch Laboratories, Inc. (West Grove, PA, USA). Glyceraldehyde-3-phosphate dehydrogenase (GADPH) was purchased from Ruierkang Biotech Co. Ltd. (Tianjin, China). Rabbit anti-I*κ*Ba antibody and anti-NF-κB p65 antibody utilized for western blotting and immunofluorescence were purchased from Abcam (Cambridge, UK) and CST (Boston, USA), respectively. Anti-Bcl-2 antibody and anti-Bax antibody were purchased from Abcam (Cambridge, UK). Non-specific rabbit polyclonal antibody and fluorescein isothiocyanate (FITC)-conjugated goat anti-rabbit IgG were purchased from Wuhan Humei Biotech Co., Ltd. (Wuhan, China). TRIzol reagent was obtained from Tiangen Biotech Co., Ltd. (Beijing, China). PrimeScript™ room temperature (RT) reagent kit with gDNA Eraser, SYBR® Premix Ex Taq™ II, ROX plus, and DL2000 DNA marker was purchased from TaKaRa Baoshengwu (China). Electrochemiluminescence (ECL) was obtained from Pierce (Wisconsin, USA).

### Cell Culture

APP/PS1-HEK293 cells were purchased from Hanbio (Shanghai, China). The cells were cultured in a DMEM medium containing 10% fetal bovine serum, 1% penicillin/streptomycin at 37℃ under 5% CO_2_, and culture media was replaced every 3 days. After the cell line reached a confluence of 70–80%, the cells were seeded on 96-well culture plates at a density of 1.5 × 10^5^/mL and stimulated with ginkgolide (Baiyu®) in the absence or presence of different concentrations (100 μg/ml, 200 μg/ml, 300 μg/ml, and 400 μg/ml), and detection was carried out at 0 h, 24 h, 48 h, and 72 h post-treatment. Briefly, screening via CCK-8 assay was performed to ascertain the optimal time point and concentration for cell proliferation as follows: (1) After removing the DMEM, 100 μL CCK-8 solution per well was added into the culture plates at a concentration of 10%, and the plates were incubated for 2 h in a 37℃ incubator; (2) The optical density of each well was then determined using a fully automatic multifunctional microplate reader (wavelength: 450 nm). The drug concentration range was further reduced to 25 μg/ml, 50 μg/ml, 75 μg/ml, 100 μg/ml, 125 μg/ml, and 150 μg/ml for observation to obtain additional confirmation of the optimal concentration. Based on these experiments, an *in vitro* study was carried out using untreated APP/PS1-HEK293 as the control group, while APP/PS1-HEK293 cells were treated with high-dosage (100 μg/ml) and low-dosage (50 μg/ml) ginkgolide, as well as its components ginkgolide B (100 μg/ml) and bilobalide (100 μg/ml) formed the interventional groups. After 48 h of co-culture, cells were harvested on the third day for further analysis.

### Quantitative Reverse Transcription–Polymerase Chain Reaction (PCR)

Total ribonucleic acid (RNA) was extracted from APP/PS1-HEK293 cells in each group using TRIzol reagent. Following the manufacturer’s instructions, 2 μl of RNA was reverse transcribed into cDNA using a PrimeScript™ RT reagent kit with a gDNA Eraser. PCRs were performed in a total reaction volume of 20 μl of 2 μl cDNA template, 10 μl Master Mix (2×), 0.5 μl of each primer, and 7 μl of distilled deionized water. The primers were synthesized by Invitrogen, USA. For NF-*κ*B p65: forward primer 5’-CTGCAGTTTGATGATGAAGA-3’, reverse primer 5’-TAGGCGAGTTATAGCCTCAG-3’; for I*κ*Ba: forward primer 5’-TGGT GTCCTTGGGTGCTG-3’, reverse primer 5’-GCTGTATCCGGGTGCTTG-3’; for bcl-2: forward primer 5’-GCCTTCTTTGAGTTCGGTGGG-3’, reverse primer 5’-GCCGGTTC AGGTACTCAGTCATC-3’; for Bax: forward primer 5’-GACGAACTGGACAGTAACAT GGAGCT-3’, reverse primer 5’-CGGCCCCAGTTGAAGTTGC-3’; for β-actin: forward primer 5’-ACTTAGTTGCGTTACACCCTT-3’, reverse primer 5’-GTCACCTTCACCGTT CCA-3’. Real-time PCR was done in an ABI 7500Fast Sequence Detector. All amplifications were carried out in triplicate for each sample. Amplifications were performed at 95℃ for 30 s, followed by 40 cycles of 95℃ for 5 s and 60℃ for 40 s. The 2–ΔΔCT (comparative threshold cycle or CT) method was then applied to calculate the mRNA expression levels of each gene, as described by the manufacturer (Technical Bulletin 2; Applied Biosystems).

### Western Blotting

The cells from the control and interventional groups were disposed of in the culture dish. Total protein was extracted by cell lysis and measured using a BCA assay kit (Sigma, St. Louis, USA) and stored at −80℃ until use. Briefly, 13 μg of total protein/well was separated on a 12% polyacrylamide gel and transferred onto a nitrocellulose membrane (Amersham, UK). The membrane was blocked for 1 h using 3% BSA, TBS-Tween-20 (TTBS), 0.2% azide at pH 7.4, and washed three times by TTBS containing 50 mM Tris, 0.5 M NaCl, 0.05% Tween 20 at pH 7.4. It was then hybridized with rabbit anti-I*k*Ba antibody, anti-NF-*κ*B p65 antibody, anti-Bcl-2 antibody, or anti-Bax antibody, as well as an anti-GAPDH antibody (1:10000 dilution) as an internal control overnight at 4℃, followed by incubation with HRP-conjugated goat anti-rabbit IgG (1:20000 dilution) for 30 min at 25℃. After washing six times with TTBS, 3 ml ECL was added to the membrane for 3–5 min at 25℃. The membrane was exposed for 10 s to 5 min, and the integrated density value was calculated as above. The gray value of each band was analyzed using image software G, and semi-quantitative analysis of protein expression in each group was carried out using an independent normalization method.

### Immunofluorescence

The cells were fixed in 4% paraformaldehyde solution and blocked with 5% bovine serum albumin (BSA) and then incubated with primary antibodies including rabbit anti-IkBa antibody, anti-NF-*κ*B p65 antibody, anti-Bcl-2 antibody, or anti-Bax antibody, as well as non-specific rabbit polyclonal antibody as control antibody overnight at 4℃, followed by a 1-h incubation with FITC-conjugated goat anti-rabbit IgG at a dilution of 1:100 in PBS at 37℃. Images using the ImageView software were acquired with an Olympus fluorescent microscope (CKX41) after staining of nuclei with propidium iodide (10 µg/ml) for 10 min.

### ELISA

Supernatant levels of TNF-α, IL-1β, and IL-6 were measured using an ELISA kit according to the manufacturer’s instructions. All assays were performed in a blinded manner.

### Statistical Analysis

Statistical analysis was performed using GraphPad Prism 8 (GraphPad Software Inc., San Diego, CA, USA). All data are presented as the mean ± standard deviation (proliferative activities by CCK-8 assay; supernatant TNF-α, IL-1β, and IL-6; NF-*κ*B p65, I*κ*Ba, Bcl-2, and Bax mRNA). Data were analyzed by one-way analysis of variance (ANOVA) with the Student–Newman–Keuls post hoc test. Statistical significance was set at *P* < 0.05.

## Results and Discussion

### Effects of Ginkgolide on Cell Proliferation

**Fig. 1:**
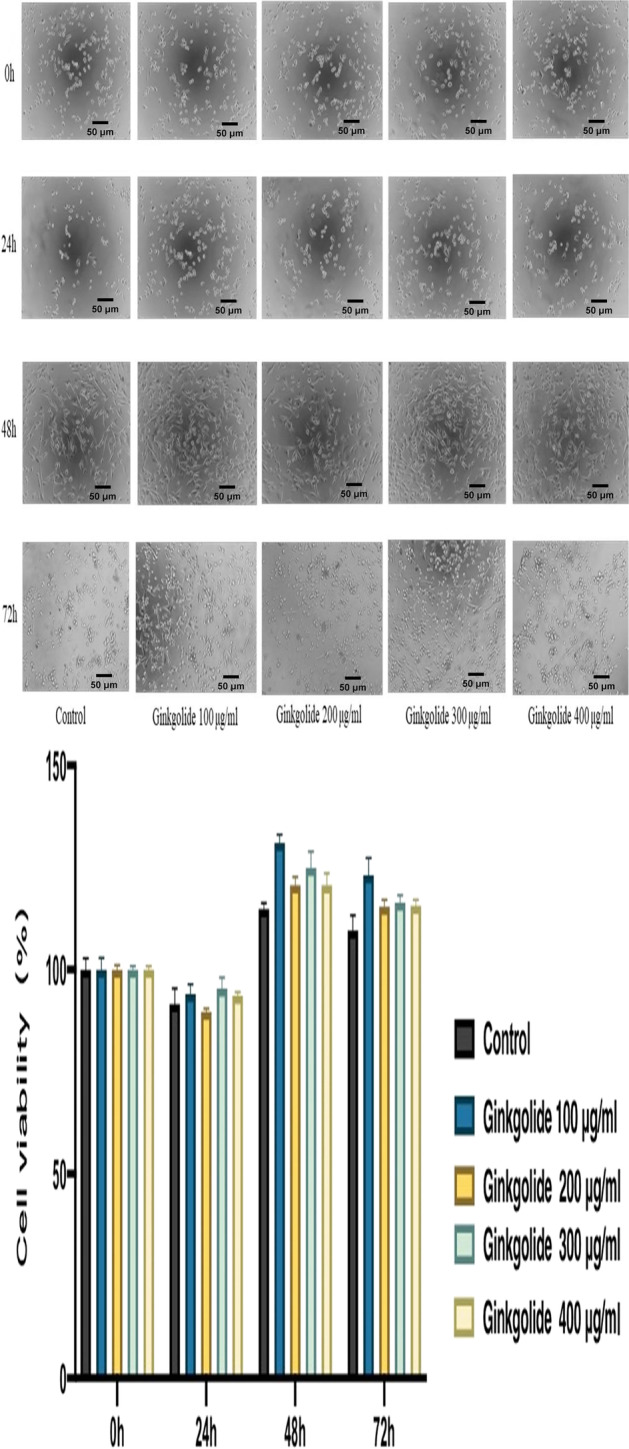
Effects of different dosages of ginkgolide on cell proliferative activity at different time points. Cell viability was measured by a cell counting kit-8 (CCK-8) assay. APP/PS1-HEK-293 cells were treated with different dosages of ginkgolide (0 μg/ml, 100 μg/ml, 200 μg/ml, 300 μg/ml, and 400 μg/ml) for 0 h, 24 h, 48 h, and 72 h, respectively, and then the cells were observed with an inverted microscope (100×)

**Fig. 2:**
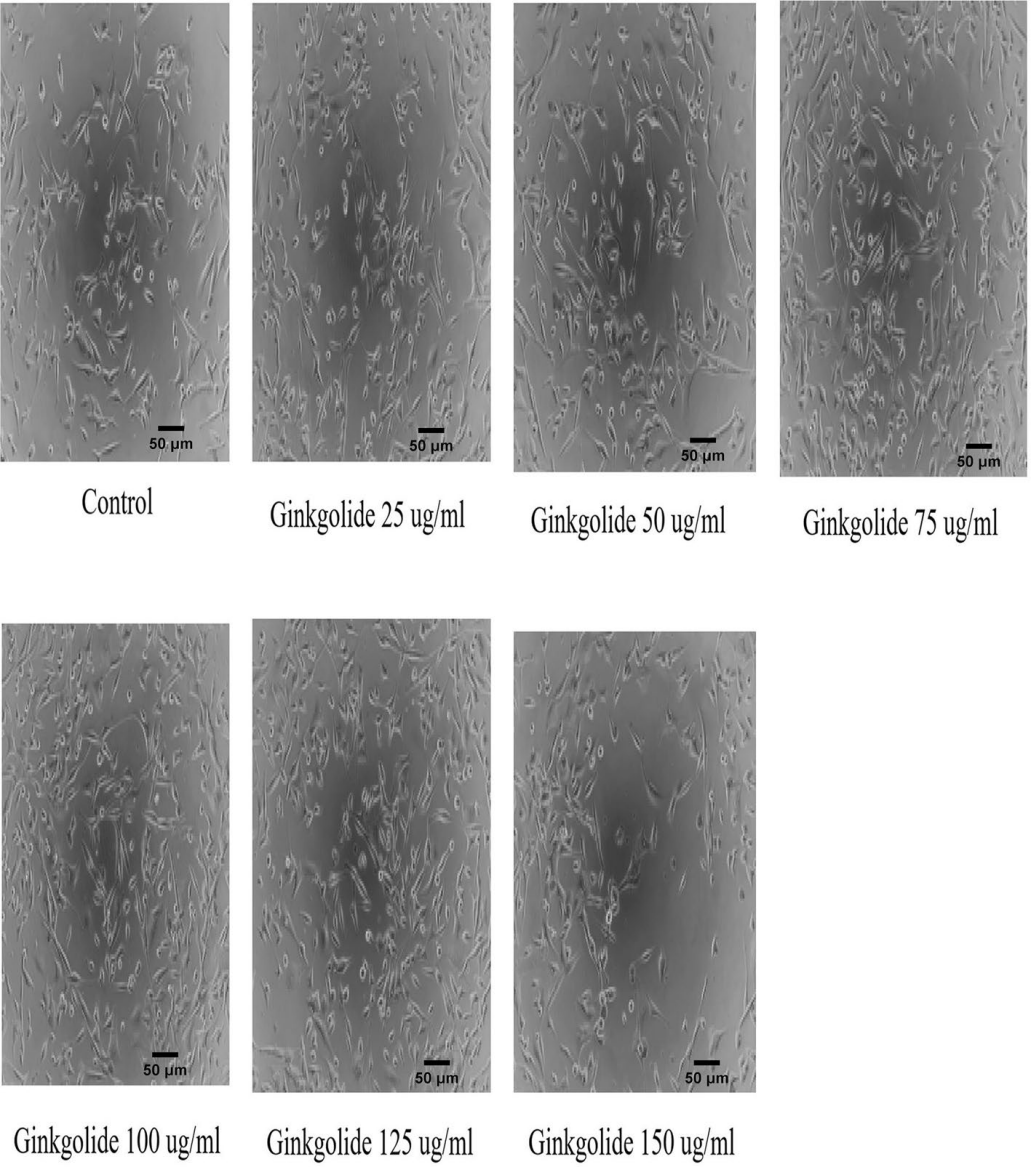
Effects of different dosages of ginkgolide on cell proliferative activity at 48 h post-treatment. APP/PS1-HEK-293 cells were treated with different dosages of ginkgolide (25 μg/ml, 50 μg/ml, 75 μg/ml, 100 μg/ml, 125 μg/ml, and 150 μg/ml) for 48 h, and then the cells were observed with an inverted microscope (100×)

The proliferative activities associated with each dosage group (100 μg/ml, 200 μg/ml, 300 μg/ml, and 400 μg/ml) demonstrated a downward-upward-downward trend after 24 h, 48 h, and 72 h of treatment with ginkgolide, and cell viability at 48 h post-treatment was significantly higher than that at 0 h and 24 h post-treatment (*P* < 0.01). Furthermore, at 48 h post-treatment, the cell viability at different dosages was significantly increased compared to the control group, particularly at a concentration of 100 μg/ml (Table 1 and Fig. 1). Based on the above findings, 48 h was selected as the best time point for promoting cell proliferation, and the drug dose range (25 μg/ml, 50 μg/ml, 75 μg/ml, 100 μg/ml, 125 μg/ml, and 150 μg/ml) was further narrowed down to determine the optimal dosage. As a result, the different dosage groups exhibited a marked increase in cell viability compared to the control group (*P* < 0.01, *P* < 0.05), especially at the concentration of 100 μg/ml (Table 2 and Fig. 2). Finally, 100 μg/ml and 48 h post-treatment with ginkgolide were chosen as the optimal concentration and time point for cell proliferation.

**Table 1:** Effects of different doses of ginkgolide on proliferative activities at different time points

	0 h (%)	24 h (%)	48 h (%)	72 h (%)
Control group (*n* = 3)	100 ± 2.74	91.55 ± 3.84	114.88 ± 1.44**	109.55 ± 3.70
100 μg/ml (*n* = 3)	100 ± 2.86	94.09 ± 2.35	131.06 ± 1.97**^##^	123.14 ± 4.27
200 μg/ml (*n* = 3)	100 ± 1.13	89.70 ± 0.81	120.65 ± 2.07**	115.42 ± 1.62
300 μg/ml (*n* = 3)	100 ± 0.83	95.41 ± 2.64	124.92 ± 4.03**	116.43 ± 1.74
400 μg/ml (*n* = 3)	100 ± 0.92	93.56 ± 0.96	120.60 ± 3.00**	115.59 ± 1.47

^**^P < 0.01, compared with cell viability at 0 h and 24 h post-treatment indicated by post hoc test

^##^*P* < 0.01, compared with the control group at 48 h post-treatment indicated by post hoc test

**Table 2:** Effects of different doses of ginkgolide on cell proliferative activities at 48 h post-treatment

	Cell viability (%)	*P value*	*P value*
Control group (*n* = 3)	107.41 ± 3.79	-	<0.0001**
25 μg/ml (*n* = 3)	125.10 ± 0.60	0.0010**	0.0114*
50 μg/ml (*n* = 3)	125.85 ± 3.99	0.0040**	0.0194*
75 μg/ml (*n* = 3)	119.55 ± 2.52	0.0100*	0.0003**
100 μg/ml (*n* = 3)	135.06 ± 2.45	<0.0001**	-
125 μg/ml (*n* = 3)	124.37 ± 2.76	0.0030**	0.0068**
150 μg/ml (*n* = 3)	122.21 ± 5.08**	0.0160*	0.0015**

^**^P < 0.01 or *P < 0.05, compared with ginkgolide (100 μg/ml) indicated by post hoc test

### Intracellular mRNA Expression Levels of NF-κB p65, IκBa, Bcl-2, and Bax

**Fig. 3:**
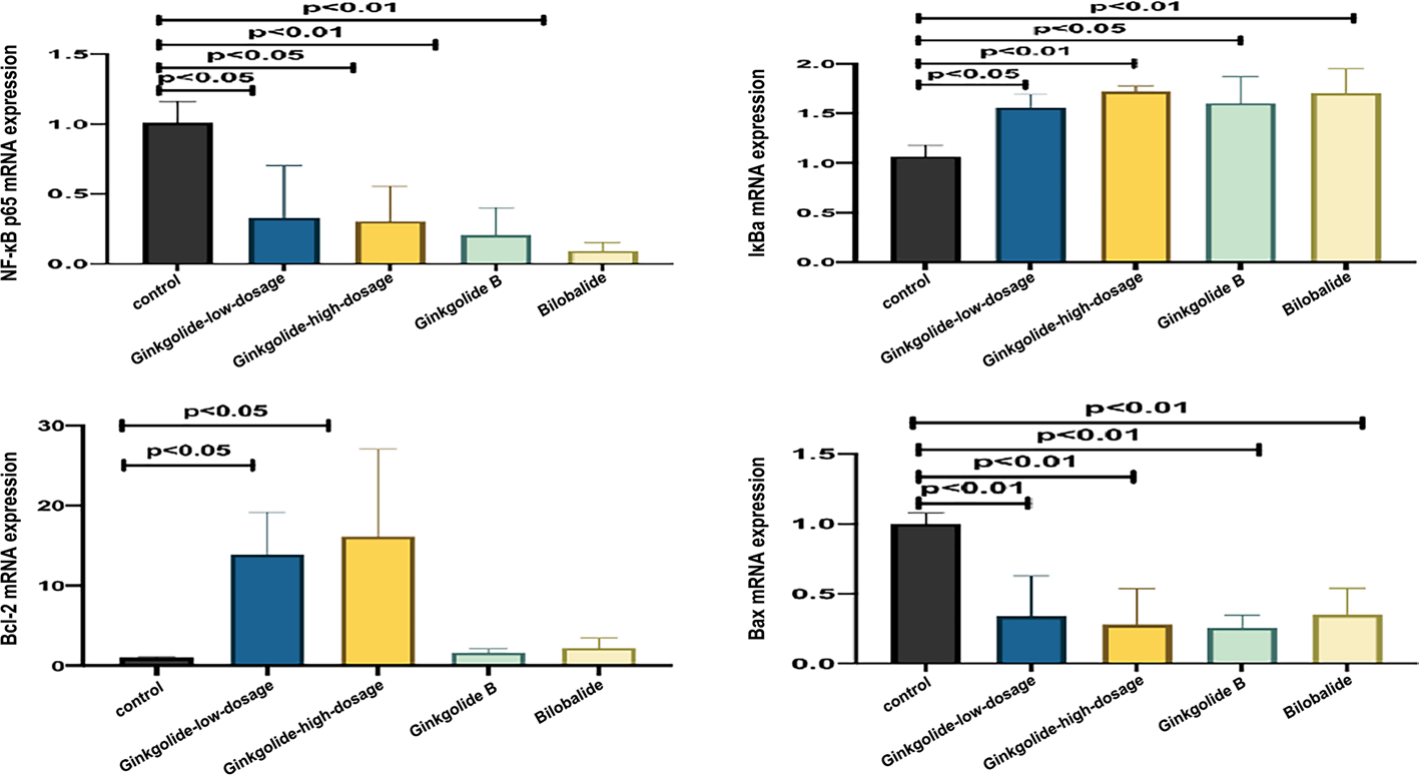
Effects of different doses of ginkgolide and its components (ginkgolide B and bilobalide) in the mRNA expression levels of NF-*κ*B p65, I*κ*Ba, Bcl-2, and Bax

Compared with the control group, there was a decrease in the intracellular mRNA expression levels of NF-*κ*B p65 and Bax (*P* < 0.01, *P* < 0.05), but an increase in the mRNA expression levels of I*κ*Ba (*P* < 0.01 and *P* < 0.05) in both high- and low-dosage ginkgolide-, ginkgolide B-, and bilobalide-treated groups, as well as an increase in the mRNA expression levels of Bcl-2 in the high- and low-dosage ginkgolide-treated groups (*P* < 0.05) (Table 3 and Fig. 3).

**Table 3:** Effects of different doses of ginkgolide and its components (ginkgolide B and bilobalide) on the intracellular expression of NF-*κ*B p65, I*κ*Ba, bcl-2, and Bax mRNA

	NF-*κ*B p65	I*κ*Ba	bcl-2	Bax
Control group (*n* = 3)	1.008 ± 0.153	1.064 ± 0.053	1.002 ± 0.074	1.002 ± 0.756
Low-dosage ginkgolide (*n* = 3)	0.328 ± 0.373*	1.558 ± 0.005*	11.264 ± 7.626*	0.339 ± 0.288**
High-dosage ginkgolide (*n* = 3)	0.302 ± 0.252*	1.721 ± 0.060**	16.110 ± 10.870*	0.280 ± 0.254**
Ginkgolide B (*n* = 3)	0.207 ± 0.195**	1.603 ± 0.306*	1.664 ± 0.442	0.260 ± 0.086**
Bilobalide (*n* = 3)	0.092 ± 0.061**	1.701 ± 0.306**	2.168 ± 1.290	0.351 ± 0.189**

^**^P < 0.01 or *P < 0.05, compared with the control group indicated by post hoc test

### Intracellular Protein Expression Levels of NF-κB p65, IκBa, Bcl-2, and Bax

**Fig. 4:**
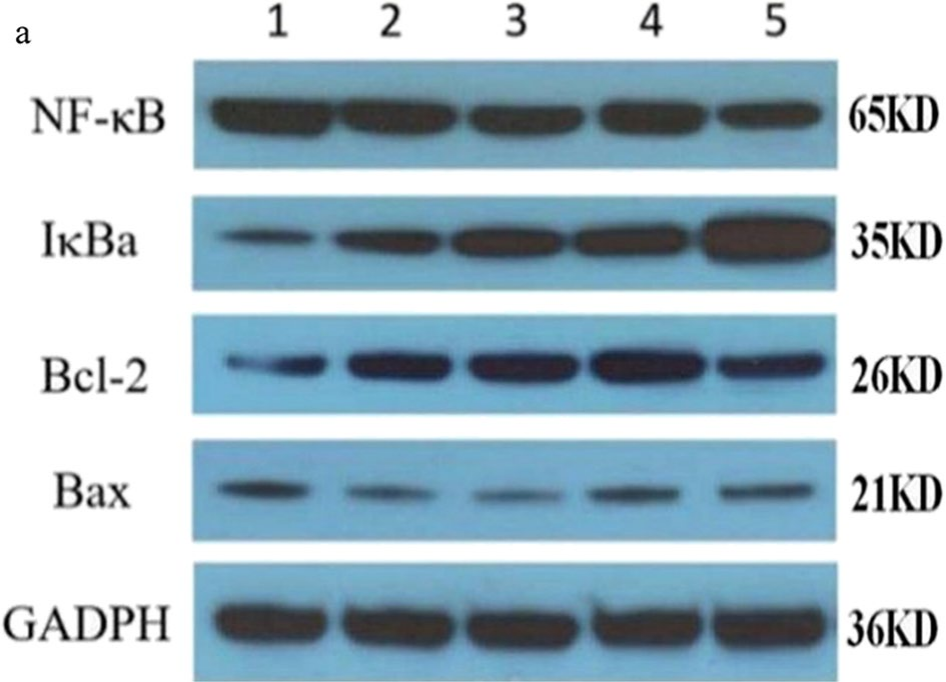

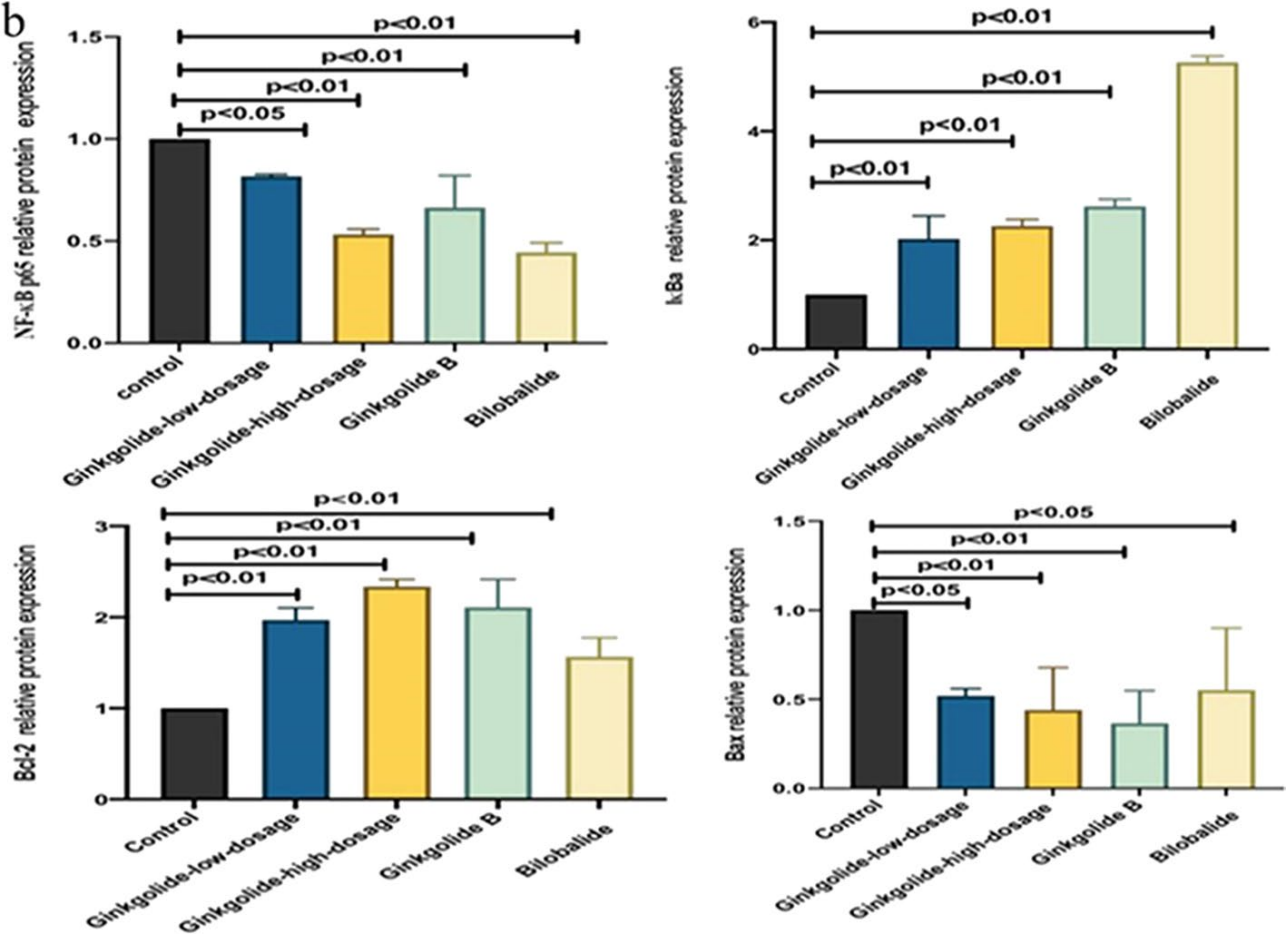
Detection of intracellular protein expression of NF-*κ*B p65, I*κ*Ba, Bcl-2, and Bax by Western blotting. (**a**) Western blotting. Lane 1, control group; lane 2, low-dosage ginkgolide; lane 3, high-dosage ginkgolide; lane 4, ginkgolide B; lane 5, bilobalide; (**b**) Effects of different doses of ginkgolide and its components (ginkgolide B and bilobalide) on intracellular protein expression of NF-*κ*B p65, I*κ*Ba, Bcl-2, and Bax

**Fig. 5:**
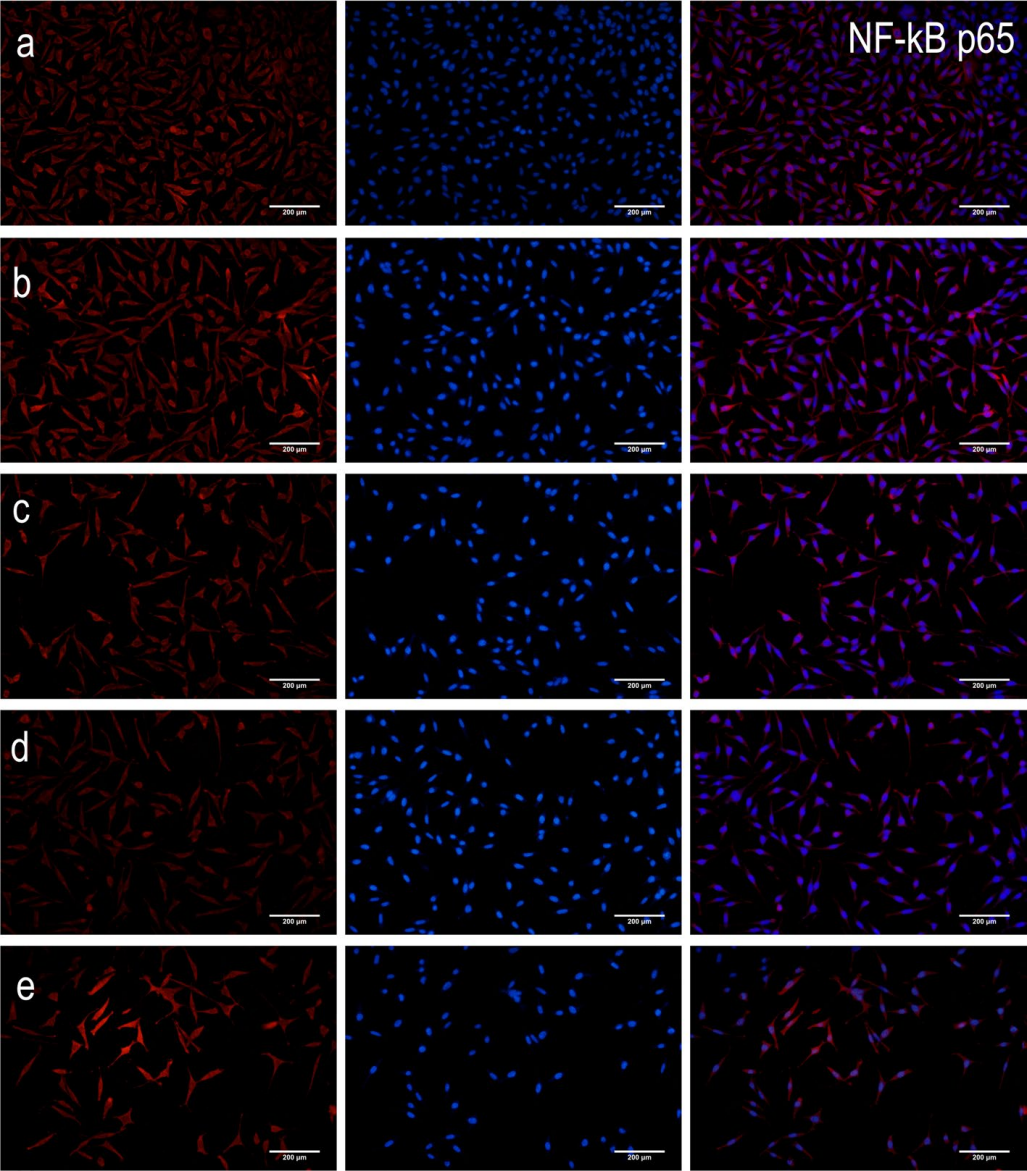

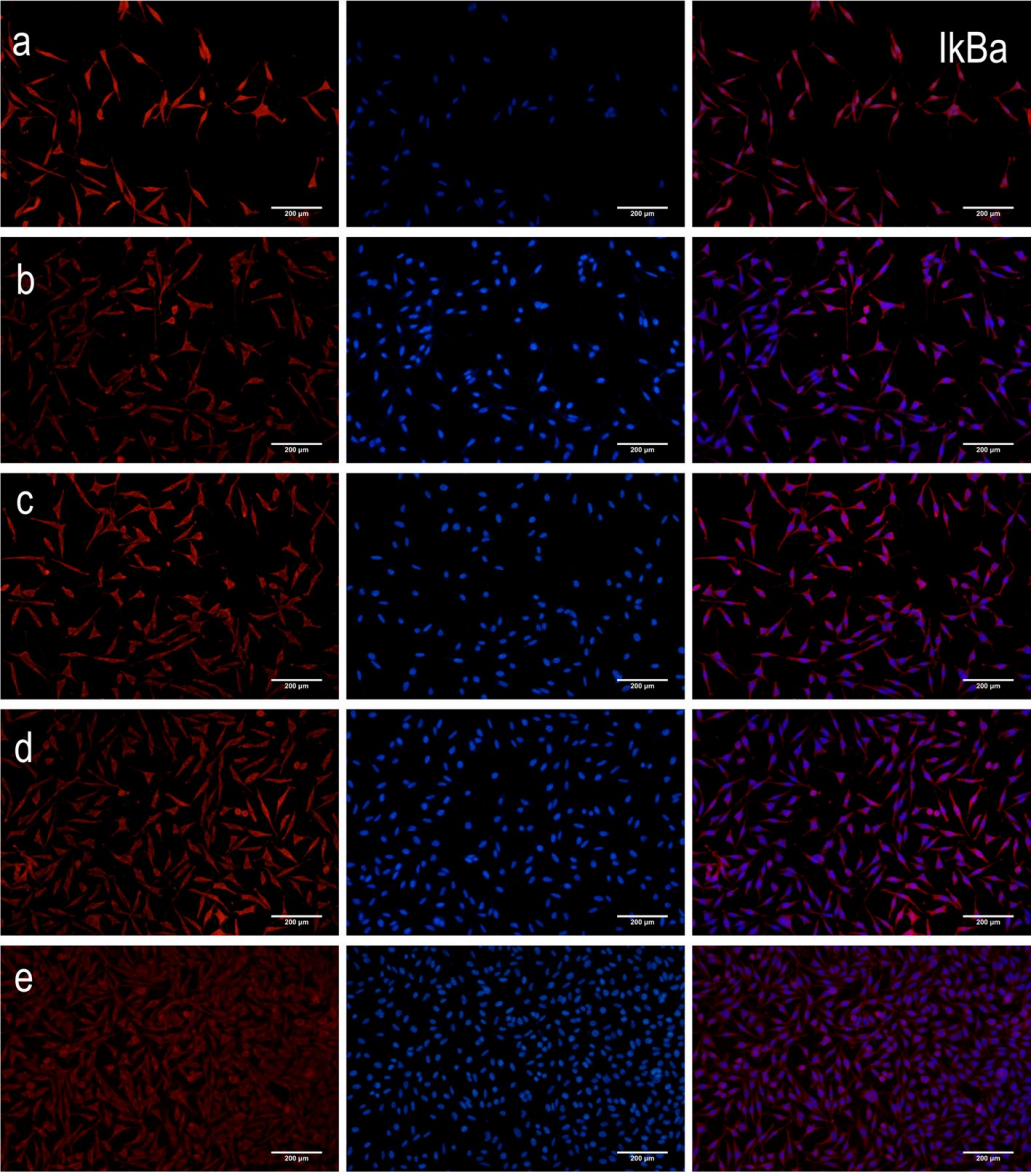

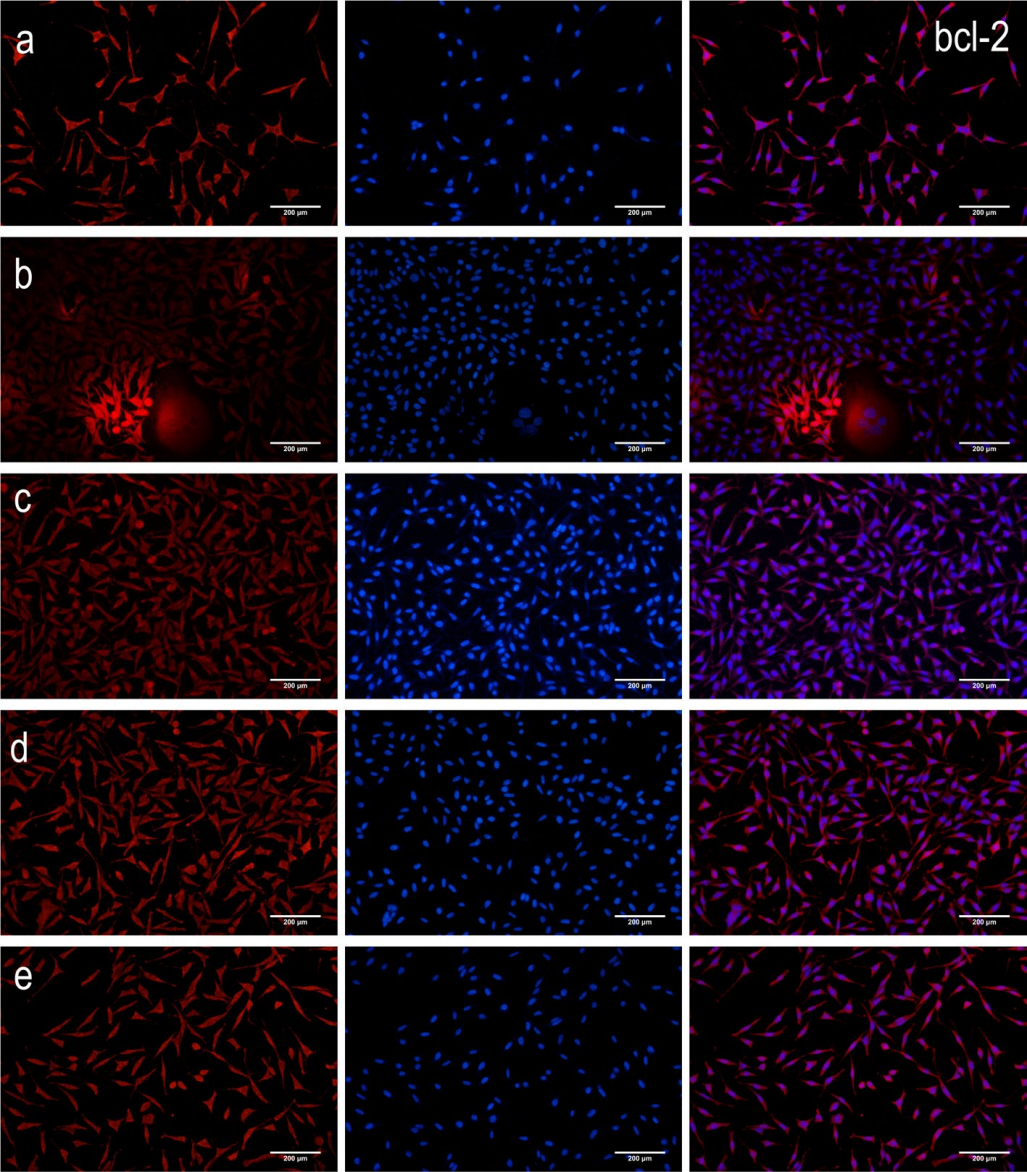

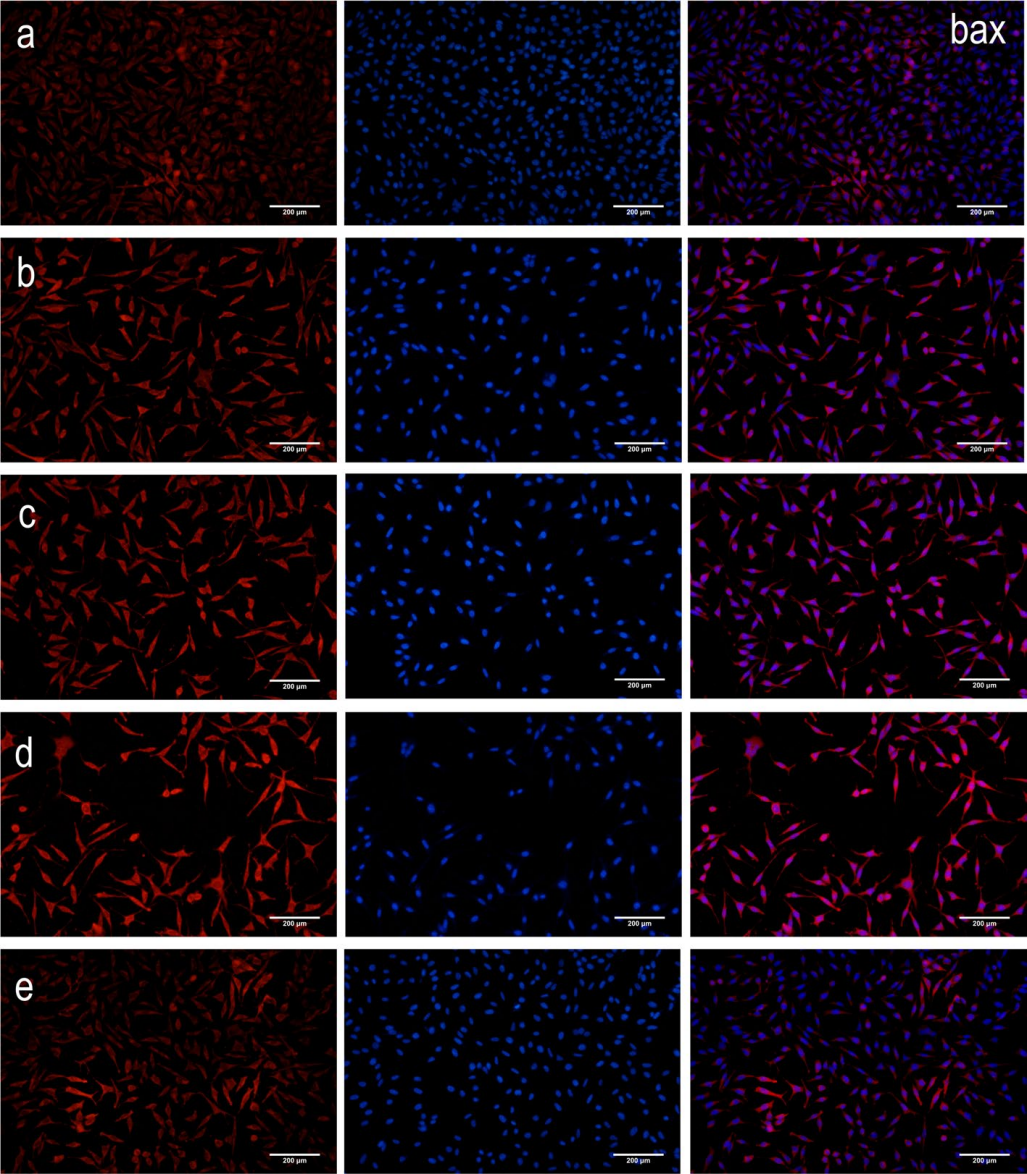
Detection of protein expression of NF-*κ*B p65, I*κ*Ba, Bcl-2, and Bax by immunoflourescent staining of APP/PS1-HEK-293 cells. Blue coloration indicates NF-κB p65, IκBa, Bcl-2, or Bax staining, while red coloration represents nuclear staining: (**a**) control group, (**b**) low-dosage (50 μg/ml) ginkgolide, (**c**) high-dosage (100 μg/ml) ginkgolide, (**d**) ginkgolide B (100 μg/ml), and (**e**) bilobalide (100 μg/ml)

Compared to the control group, there was a decrease in the intracellular protein expression levels of NF-*κ*B p65 and Bax (*P* < 0.01 and *P* < 0.05), but an increase in the protein expression levels of I*κ*Ba and Bcl-2 (*P* < 0.01) in both high- and low-dosage ginkgolide-, ginkgolide B-, and bilobalide-treated groups (Fig. 4). In parallel with the above-mentioned alternations, remarkable differences were found among HEK 293 cells expressing NF-*κ*B p65, I*κ*Ba, Bcl-2, and Bax (Fig. 5).

### Supernatant TNF-α, IL-1β, and IL-6 Levels

**Fig. 6:**
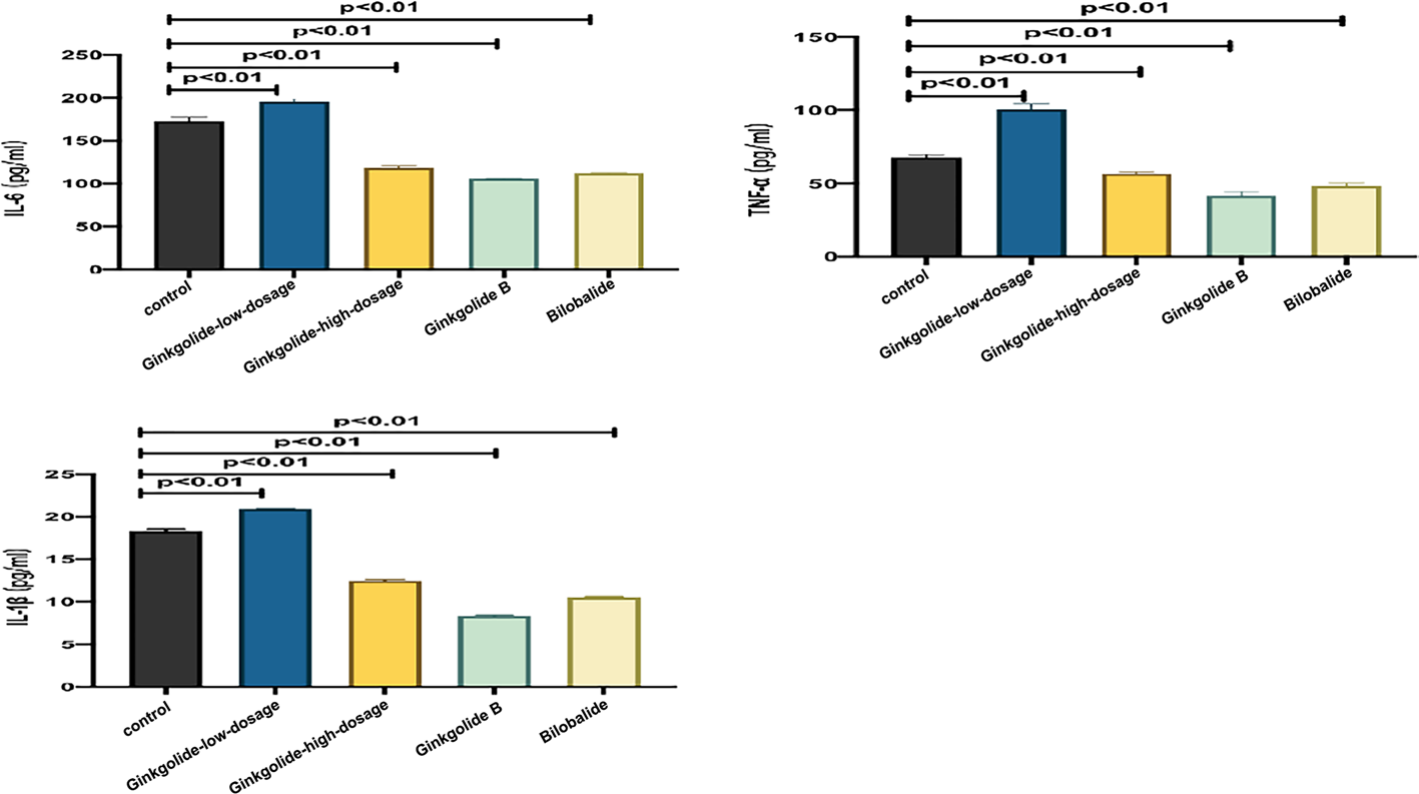
Detection of the supernatant levels of TNF-α, IL-1β, and IL-6 in different doses of ginkgolide and its components (ginkgolide B and bilobalide)

The supernatant levels of TNF-α, IL-1β, and IL-6 in the high-dosage (100 μg/ml) ginkgolide-, ginkgolide B-, and bilobalide-treated groups were lower than those in the control group (*P* < 0.01), but higher in the low-dosage (50 μg/ml) ginkgolide-treated group (*P* < 0.01) (Table 4 and Fig. 6).

**Table 4:** Effects of different doses of ginkgolide and its components (ginkgolide B and bilobalide) on supernatant levels of TNF-α, IL-1β, and IL-6

	TNF-α (pg/ml)	IL-1β (pg/ml)	IL-6 (pg/ml)
Control group (*n* = 3)	67.78 ± 2.05**	18.31 ± 0.24**	172.75 ± 4.42**
Low-dosage ginkgolide (*n* = 3)	100.54 ± 3.82**	20.89 ± 0.06**	195.77 ± 2.71**
High-dosage ginkgolide (*n* = 3)	57.81 ± 3.74**	12.45 ± 0.19**	118.43 ± 2.08**
Ginkgolide B (*n* = 3)	41.37 ± 2.93**	8.29 ± 0.16**	105.92 ± 0.32**
Bilobalide (*n* = 3)	48.42 ± 1.68**	10.48 ± 0.16**	111.96 ± 0.67**

^**^P < 0.01, compared with the control group indicated by post hoc test

In our study, APP/PS1-HEK293 cells showed markedly increased proliferative activity at 48 h post-treatment with ginkgolide, as well as a lower and higher expression or intracellular accumulation of NF-*κ*B p65 and I*κ*Ba at transcript and protein levels, respectively, indicating that ginkgolide could improve cell viability by suppressing the activation of intracellular NF-*κ*B signaling. Furthermore, component analysis of ginkgolide revealed that ginkgolide B downregulated the production of NF-*κ*B p65, while bilobalide upregulated the expression of I*κ*Bα at both transcript and protein levels. In parallel with the altered intracellular accumulation of NF-*κ*B p65 and I*κ*Bα, our results suggest that these two components might exert distinctive regulatory effects on the NF-*κ*B signaling pathway. Nevertheless, future research is required to determine their exact roles.

Aβ accumulation can accelerate neuronal apoptosis, although the exact molecular mechanism remains uncertain. It is well known that Aβ-induced neuronal apoptosis causing neuronal loss plays a critical role in the development of AD [22, 23]. Intriguingly, a recent study showed that Aβ1-42 could lead to cerebral vascular damage by prompting oxidative stress, inducing mitochondrial dysfunction and apoptosis of cerebral endothelial cells in a mouse model of AD [24], suggesting that the inhibition of apoptosis is beneficial for halting the progression of AD. Notably, Xiao et al. recently reported that Aβ25-35-induced apoptosis was attenuated by ginkgolide B via the upregulation of brain-derived neurotrophic factors when the cells were subjected to Aβ25-35 insult [25]. Another study reported by Wang et al. showed that ginkgolide B was able to protect rat astrocytes from Aβ1-42-induced apoptosis by inhibiting endoplasmic reticulum stress, oxidative stress, and Aβ1-42 production, possibly through the activation of the AMPK pathway [26]. Interestingly, Yin et al. found that in the Aβ25-35-induced rat model of AD, bilobalide significantly reduced the neuronal damage and apoptosis in the frontal cortex and hippocampus CA1 [27]. In line with this finding, our study showed that ginkgolides downregulated the expression of the apoptosis protein Bax and upregulated the expression of the anti-apoptotic protein Bcl-2 at transcript and protein levels in parallel with their altered accumulation in the nucleus, which once again validated the anti-apoptotic effects of ginkgolide in an AD cell model. In addition, ginkgolide B and bilobalide decreased the mRNA and protein levels or intracellular accumulation of Bax, but increased the mRNA and protein levels or intracellular accumulation of Bcl-2 in APP/PS1-HEK293 cells to some extent, suggesting that they may exert synergistic anti-apoptotic action against AD via multiple mechanisms.

Increasing evidence has substantiated the crucial role of the Aβ-induced inflammatory response in the neurodegenerative process of AD. Once microglia are activated by Aβ, there is a resultant secretion of proinflammatory cytokines, such as IL-6, IL-1β, and TNF-α [28], and IL-1β reduces the number of synaptic connections, resulting in synaptic degeneration and neuronal loss [29]. Aβ also induces neurotoxicity and the sustained release of neurotoxic factors leading to neurodegeneration, many of which are deemed to be microglia-derived, including TNF-α, nitrogen oxide (NO), IL-1β, and reactive oxygen species, which consequently accelerate the development of AD [28]. Several studies have confirmed that ginkgolide reduced the production of TNF-α, IL-1β, and IL-6 by suppressing oxidative stress, mitochondrial dysfunction, and endothelial dysfunction [30–33]. In keeping with these findings, our study demonstrated that high-dose ginkgolide significantly downregulated the production of TNF-α, IL-1β, and IL-6, in contrast to low-dose ginkgolide, suggesting that a dose-dependent pattern might exist in terms of the regulatory effects of ginkgolide on inflammation. Furthermore, the component analysis showed that both ginkgolide B and bilobalide lowered the production of the aforementioned inflammatory cytokines to the same extent, suggesting the existence of their synergistic anti-inflammatory effects on these cytokines.

In conclusion, we found that ginkgolide significantly enhanced the viability of APP/PS1-HEK293 cells, strongly indicative of its neuroprotective effects on AD, at least partially via suppression of the NF-*κ*B signaling pathway involving anti-apoptosis and anti-inflammation mechanisms. However, this issue needs to be further addressed by *in vivo* studies on animal models and, more importantly, human clinical trials. Nevertheless, our findings shed light on novel treatment options for AD, and ginkgolide might be a promising therapeutic agent against this disease.

## Supplementary Information

Below is the link to the electronic supplementary material.

Fig. 7 (PNG 40179 kb)

High resolution (TIF 29268 kb)

Fig. 8 (PNG 2589 kb)

High resolution (TIF 29269 kb)

Fig. 9 (PNG 27788 kb)

High resolution (TIF 29267 kb)

Fig. 10 (PNG 21399 kb)

High resolution (TIF 29267 kb)

## Data Availability

The datasets used or analyzed during the current study are available from the corresponding author on reasonable request.

## Abbreviations

Aβ: Amyloid-beta
AD: Alzheimer’s disease
APP/PS1-HEK293APP/PS1: APP/PS1 double gene-transfected HEK293 cell line
CCK-8: Cell counting kit-8
ECL: Electrochemiluminescence
ELISA: Enzyme-linked immunosorbent assay
GADPH: Glyceraldehyde-3-phosphate dehydrogenase
GB: Ginkgo biloba
IκB: Inhibitory κB
IKK: IκB kinase
IL: Interleukin
NF-κB: Nuclear factor kappa B
PCR: Polymerase chain reaction
RT: Room temperature
TNF: Tumor necrosis factor

## References

[1] Paudel YN, Angelopoulou E, Piperi C, Othman I, Aamir K, Shaikh MF (2020). Impact of HMGB1, RAGE, and TLR4 in Alzheimer’s disease (AD): From risk factors to therapeutic targeting. Cells.

[2] Cho EJ, Kim HY, Lee AY (2020). Paeoniflorin ameliorates Abeta-stimulated neuroinflammation via regulation of NF-kappaB signaling pathway and Abeta degradation in C6 glial cells. Nursing Research & Practice.

[3] Wang HM, Zhang T, Huang JK, Sun XJ (2013). 3-N-butylphthalide (NBP) attenuates the amyloid-beta-induced inflammatory responses in cultured astrocytes via the nuclear factor-kappaB signaling pathway. Cellular Physiology and Biochemistry.

[4] Lee S, Youn K, Jun M (2018). Major compounds of red ginseng oil attenuate Abeta(25–35)-induced neuronal apoptosis and inflammation by modulating MAPK/NF-kappaB pathway. Food & Function.

[5] Mitchell S, Vargas J, Hoffmann A (2016). Signaling via the NFκB system. Wiley. Interdiscip. Rev. Syst. Biol. Med..

[6] Oeckinghaus A, Hayden MS, Ghosh S (2011). Crosstalk in NF-κB signaling pathways. Nature Immunology.

[7] Wang DJ, Ratnam NM, Byrd JC, Guttridge DC (2014). NF-κB functions in tumor initiation by suppressing the surveillance of both innate and adaptive immune cells. Cell Reports.

[8] Napetschnig J, Wu H (2013). Molecular basis of NF-κB signaling. Annual Review of Biophysics.

[9] Liu F, Xia Y, Parker AS, Verma IM (2012). IKK. biology. Immunological Reviews.

[10] Sachdeva AK, Chopra K (2015). Lycopene abrogates Abeta(1–42)-mediated neuroinflammatory cascade in an experimental model of Alzheimer’s disease. Journal of Nutritional Biochemistry.

[11] Choi JY, Yeo IJ, Kim KC, Choi WR, Jung JK, Han SB, Hong JT (2018). K284–6111 prevents the amyloid beta-induced neuroinflammation and impairment of recognition memory through inhibition of NF-kappaB-mediated CHI3L1 expressionJ. Neuroinflammation.

[12] Loureiro JC, Pais MV, Stella F, Radanovic M, Teixeira AL, Forlenza OV, de Souza LC (2020). Passive antiamyloid immunotherapy for Alzheimer’s disease. Current Opinion in Psychiatry.

[13] Syed YY (2020). Sodium oligomannate: First approval. Drugs.

[14] Lakey-Beitia J, González Y, Doens D, Stephens DE, Santamaría R, Murillo E, Gutiérrez M, Fernández PL, Rao KS, Larionov OV, Durant-Archibold AA (2017). Assessment of novel curcumin derivatives as potent inhibitors of inflammation and amyloid-beta aggregation in Alzheimer’s disease. Journal of Alzheimer's Disease.

[15] El-Sayed NS, Bayan Y (2015). Possible role of resveratrol targeting estradiol and neprilysin pathways in lipopolysaccharide model of Alzheimer disease. Advances in Experimental Medicine and Biology.

[16] Zhang J, Zhen YF, Pu-Bu-Ci-Ren, Song LG, Kong WN, Shao TM, Li X, Chai XQ (2013). Salidroside attenuates beta amyloid-induced cognitive deficits via modulating oxidative stress and inflammatory mediators in rat hippocampus. Behav. Brain. Res.

[17] Ono K, Zhao D, Wu Q, Simon J, Wang J, Radu A, Pasinetti GM (2020). Pine bark polyphenolic extract attenuates amyloid-beta and tau misfolding in a model system of Alzheimer’s disease Neuropathology. Journal of Alzheimer's Disease.

[18] Achete de Souza G, de Marqui SV, Matias JN, Guiguer EL, Barbalho SM (2020). Effects of Ginkgo biloba on diseases related to oxidative stress. Planta Medica.

[19] Mohanta TK, Tamboli Y, Zubaidha PK (2014). Phytochemical and medicinal importance of Ginkgo biloba L. Natural Product Research.

[20] Kandiah N, Ong PA, Yuda T, Ng LL, Mamun K, Merchant RA, Chen C, Dominguez J, Marasigan S, Ampil E, Nguyen VT, Yusoff S, Chan YF, Yong FM, Krairit O, Suthisisang C, Senanarong V, Ji Y, Thukral R, Ihl R (2019). Treatment of dementia and mild cognitive impairment with or without cerebrovascular disease: Expert consensus on the use of Ginkgo biloba extract, EGb 761(R).*CNS*. Neurosci. Ther.

[21] Jaracz S, Malik S, Nakanishi K (2004). Isolation of ginkgolides A, B, C, J and bilobalide from G. biloba extracts. Phytochemistry.

[22] Loo DT, Copani A, Pike CJ, Whittemore ER, Walencewicz AJ, Cotman CW (1993). Apoptosis is induced by beta-amyloid in cultured central nervous system neurons. Proc Natl Acad Sci U S A.

[23] Crews L, Masliah E (2010). Molecular mechanisms of neurodegeneration in Alzheimer’s disease. Human Molecular Genetics.

[24] Fang X, Zhong X, Yu G, Shao S, Yang Q (2017). Vascular protective effects of KLF2 on Aβ-induced toxicity: Implications for Alzheimer’s disease. Brain Research.

[25] Xiao Q, Wang C, Li J, Hou Q, Li J, Ma J, Wang W, Wang Z (2010). Ginkgolide B protects hippocampal neurons from apoptosis induced by beta-amyloid 25–35 partly via up-regulation of brain-derived neurotrophic factor. European Journal of Pharmacology.

[26] Wang J, Ding Y, Zhuang L, Wang Z, Xiao W, Zhu J (2021). Ginkgolide B-induced AMPK pathway activation protects astrocytes by regulating endoplasmic reticulum stress, oxidative stress and energy metabolism induced by Aβ1-42. Molecular Medicine Reports.

[27] Yin Y, Ren Y, Wu W, Wang Y, Cao M, Zhu Z, Wang M, Li W (2013). Protective effects of bilobalide on Abeta(25–35) induced learning and memory impairments in male rats. Pharmacology, Biochemistry and Behavior.

[28] Spangenberg EE, Green KN (2017). Inflammation in Alzheimer’s disease: Lessons learned from microglia-depletion models. Brain, Behavior, and Immunity.

[29] Mishra A, Kim HJ, Shin AH, Thayer SA (2012). Synapse loss induced by interleukin-1beta requires pre- and post-synaptic mechanisms. Journal of Neuroimmune Pharmacology.

[30] Feng Z, Yang X, Zhang L, Ansari IA, Khan MS, Han S, Feng Y (2018). Ginkgolide B ameliorates oxidized low-density lipoprotein-induced endothelial dysfunction via modulating Lectin-like ox-LDL-receptor-1 and NADPH oxidase 4 expression and inflammatory cascades. Phytotherapy Research.

[31] Goldie M, Dolan S (2013). Bilobalide, a unique constituent of Ginkgo biloba, inhibits inflammatory pain in rats. Behavioural Pharmacology.

[32] Priyanka A, Nisha VM, Anusree SS, Raghu KG (2014). Bilobalide attenuates hypoxia induced oxidative stress, inflammation, and mitochondrial dysfunctions in 3T3-L1 adipocytes via its antioxidant potential. Free Radical Research.

[33] Qin YR, Ma CQ, Wang DP, Zhang QQ, Liu MR, Zhao HR, Jiang JH, Fang Q (2021). Bilobalide alleviates neuroinflammation and promotes autophagy in Alzheimer’s disease by upregulating lincRNA-p21. Am J Transl Res.

