# Supplementary figures and images for: Protective Effects of Ginkgolide on a Cellular Model of Alzheimer’s Disease via Suppression of the NF-κB Signaling Pathway
Source: Appl Biochem Biotechnol. 2022 Feb 7;194(6):2448–64. doi: 10.1007/s12010-022-03828-5 (PMC9117391; doi:10.1007/s12010-022-03828-5)

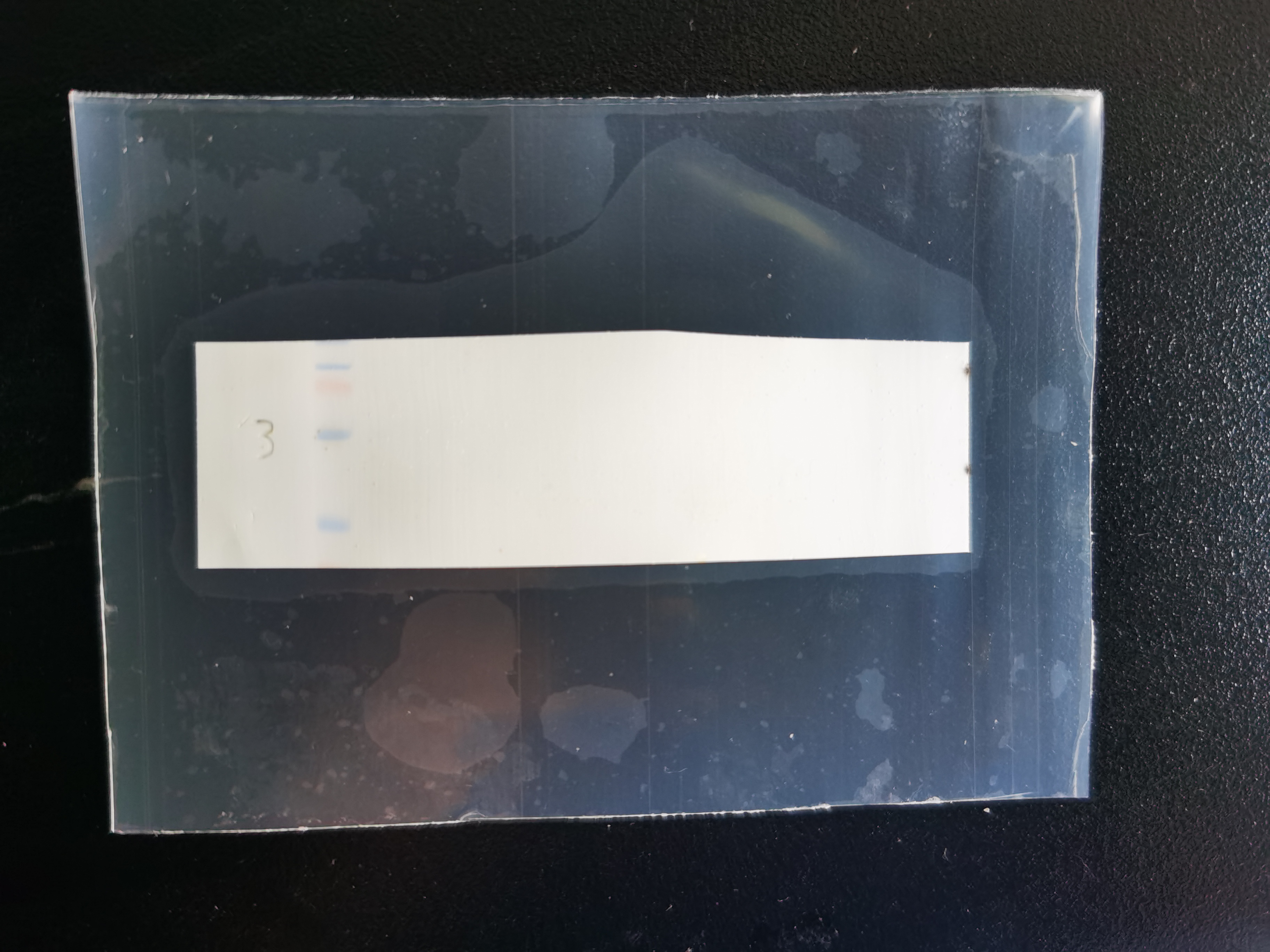

Supplement: Supplementary file 2 — High resolution (TIF 29268 kb) [file 12010_2022_3828_MOESM1_ESM.tif]

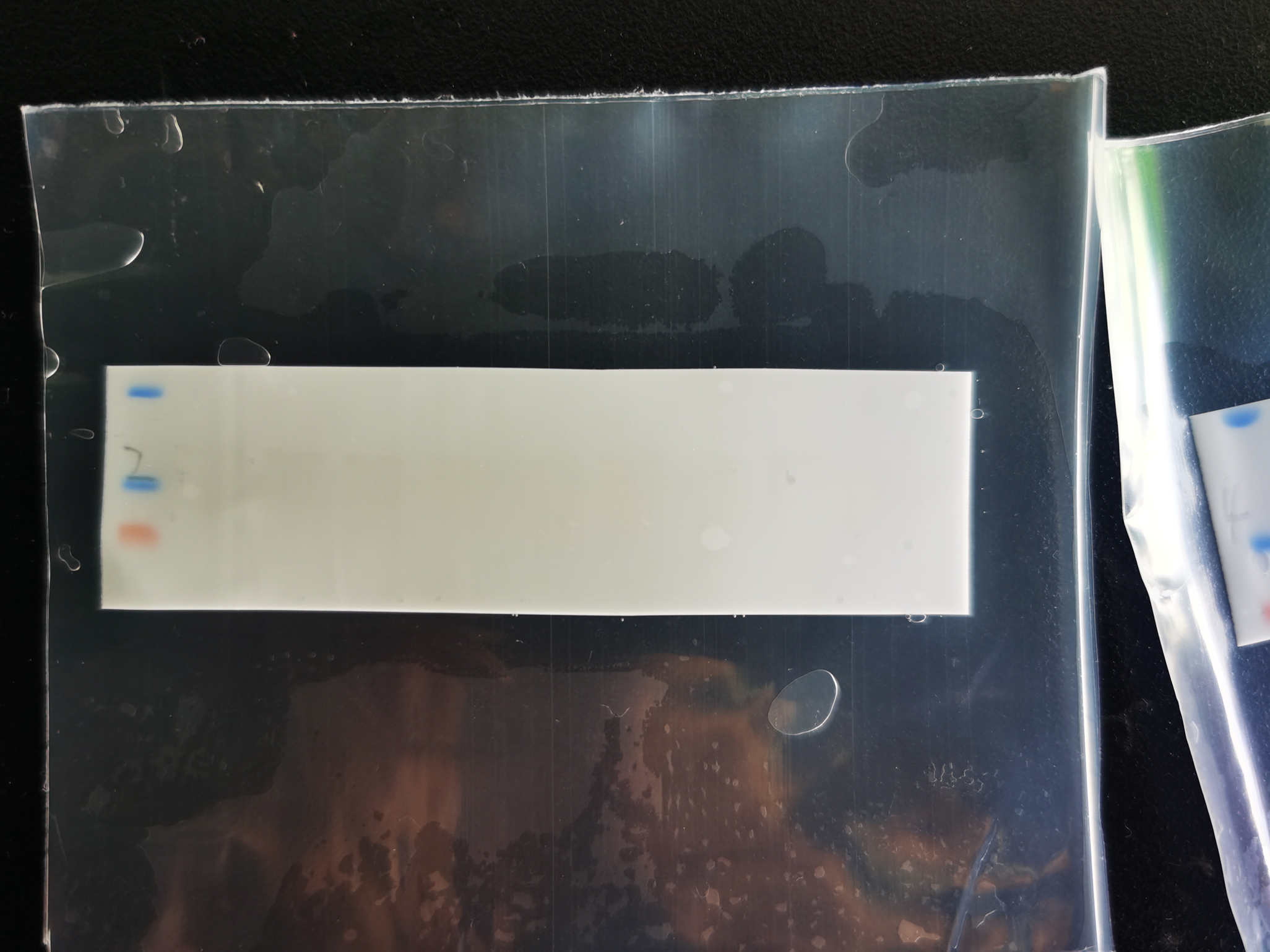

Supplement: Supplementary file 3 — (PNG 2589 kb) [file 12010_2022_3828_Fig8_ESM.png]

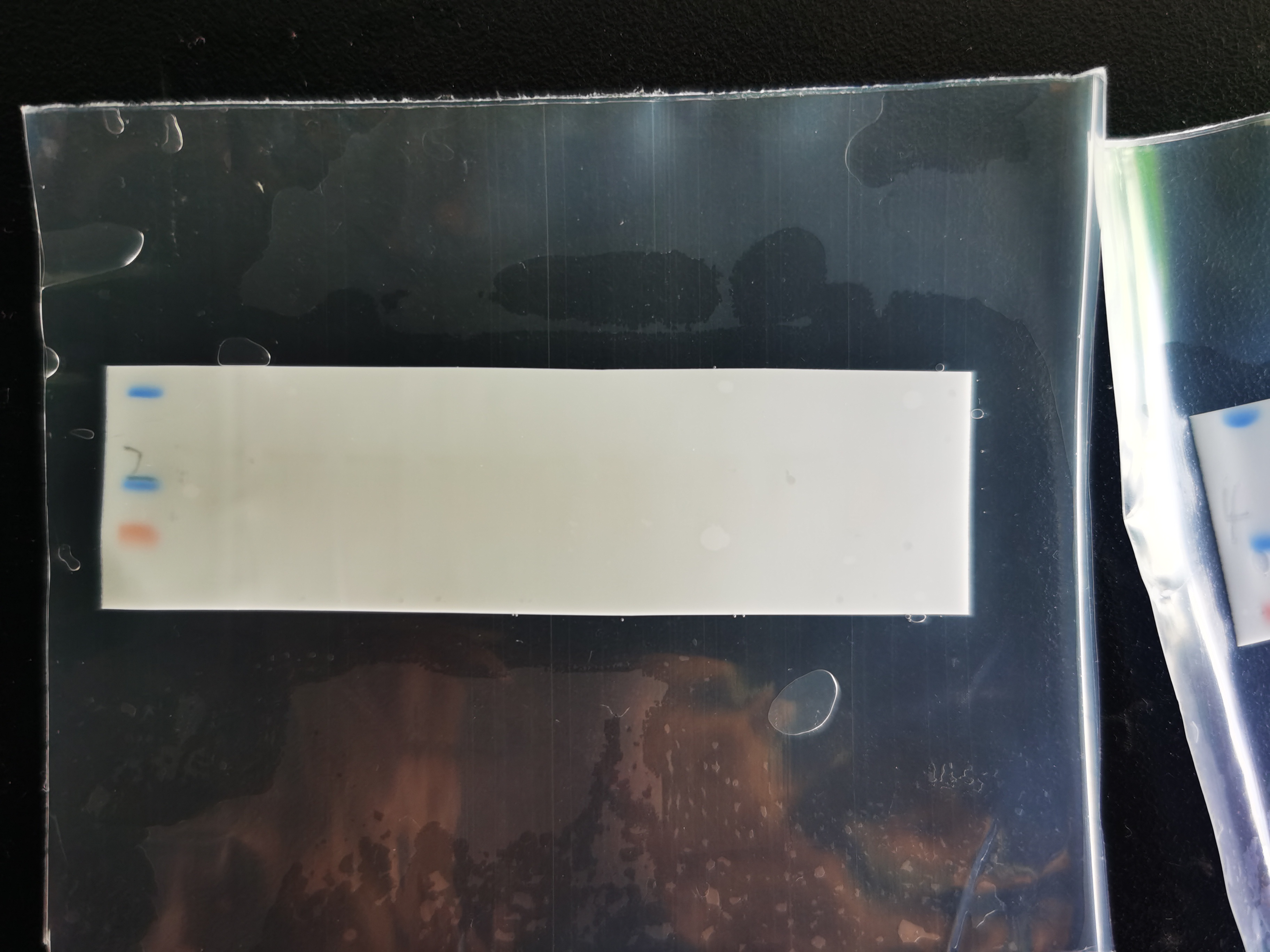

Supplement: Supplementary file 4 — High resolution (TIF 29269 kb) [file 12010_2022_3828_MOESM2_ESM.tif]

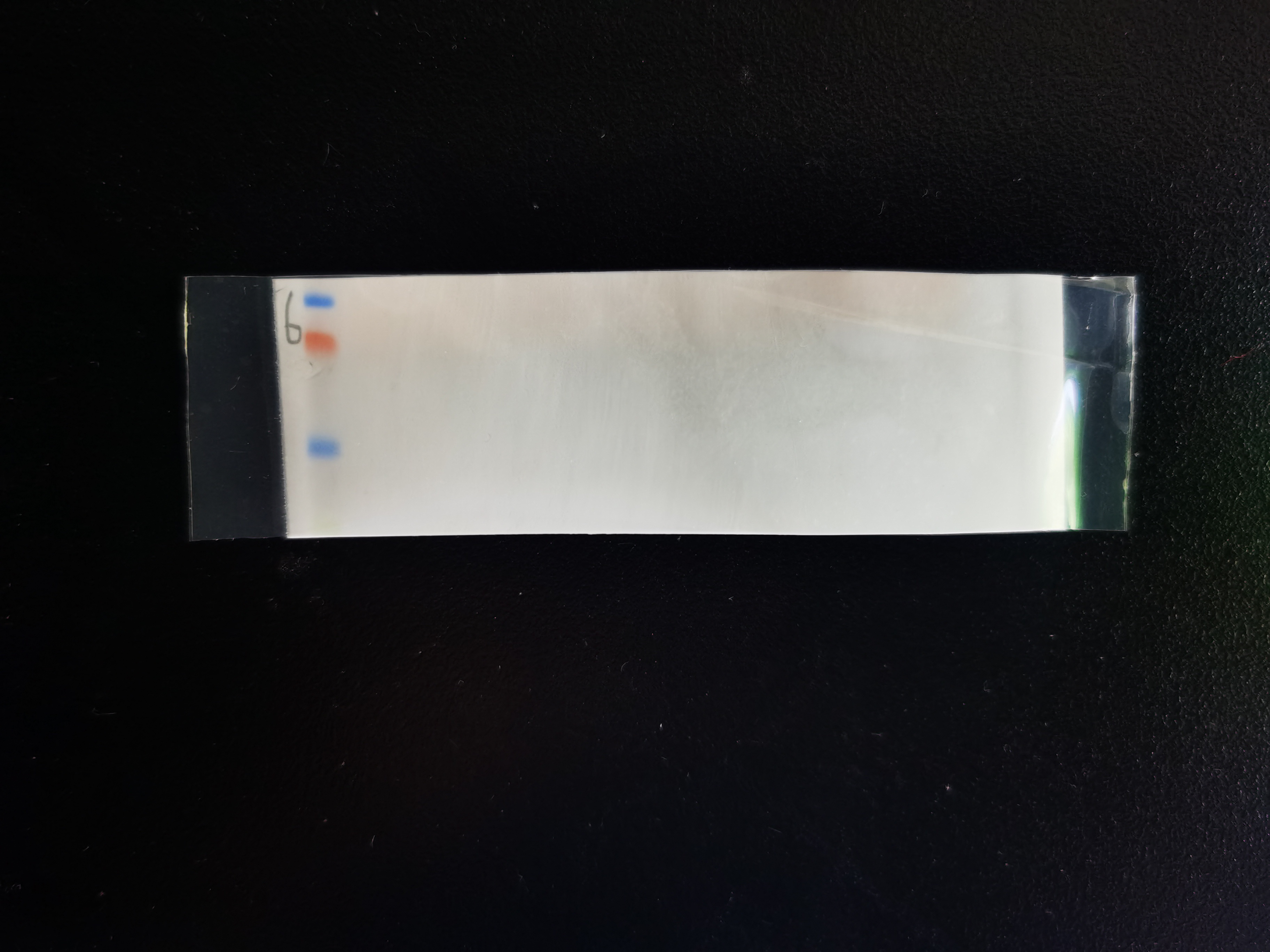

Supplement: Supplementary file 6 — High resolution (TIF 29267 kb) [file 12010_2022_3828_MOESM3_ESM.tif]

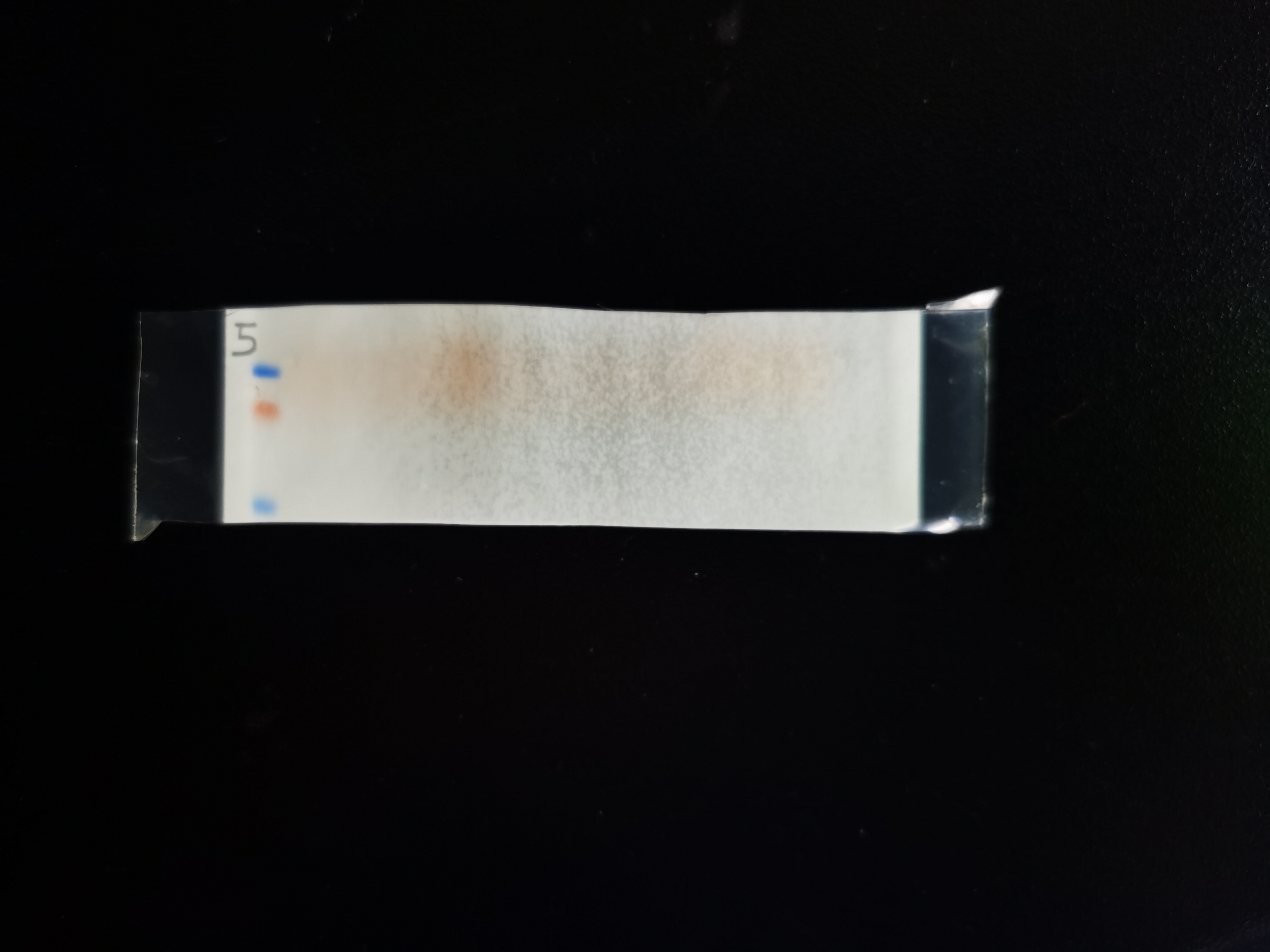

Supplement: Supplementary file 8 — High resolution (TIF 29267 kb) [file 12010_2022_3828_MOESM4_ESM.tif]
